# Intraoperative real-time fluorescence angiography with indocyanine green for evaluation of intestinal viability during surgery for an incarcerated obturator hernia: a case report

**DOI:** 10.1186/s13037-018-0173-1

**Published:** 2018-08-24

**Authors:** Dimitra Daskalopoulou, Joseph Kankam, Jens Plambeck, Peter C. Ambe, Konstantinos Zarras

**Affiliations:** 1 Department of Visceral, Minimal-Invasive and Oncological Surgery, Marien Hospital Düsseldorf, Rochusstraße 2, 40479 Düsseldorf, Germany; 2 Chair of Surgery II, Department of Medicine, Witten / Herdecke University, Witten, Germany

**Keywords:** ICG fluorescence angiography, Incarcerated obturator hernia, Intestinal blood flow, Laparoscopy

## Abstract

**Background:**

Bowel incarceration represents a dreaded complication amongst patients with hernias. The intraoperative evaluation of the bowel perfusion following hernia reduction with regard to the need for resection of ischaemic bowel can be challenging. In this case report we discuss intraoperative fluorescence angiography with indocyanine green (ICG) as an objective means of accessing bowel perfusion following hernia reduction.

**Case presentation:**

The case of a 92-year-old, caucasian, female patient presenting with symptoms of small bowel obstruction secondary to an incarcerated left sided obturator hernia is presented. An incarcerated segment of the small bowel was reduced during emergency laparoscopy. Intraoperative ICG fluorescence angiography revealed ischaemic changes in the normal appearing bowel, so that the involved segment was resected. The postoperative course was uneventful and the patient was discharged home safely on postoperative day seven.

**Conclusion:**

Intraoperative ICG fluorescence angiography provides an objective method of judging bowel perfusion and therefore represents a useful tool for assessing intestinal perfusion in patients with incarcerated hernia.

## Background

Obturator hernia defines a herniation into or through the obturator foramen. Hernia obturator accounts for just about 1% of all hernias and is truly a rare entity. Obturator hernias occur more frequently in elderly multiparous women and patients with laxity of the parietal peritoneum [1]. The clinical presentation is rather vague with non-specific symptoms. A vast majority of cases present with signs of bowel obstruction [2]. A positive Howship - Romberg sign defined by pain in the inner thigh during internal rotation of the ipsilateral hip is reported in 15–50% of cases [2]. Compression of the obturator nerve with worsening pain might be provoked via the adduction of the ipsilateral leg [3]. Physical examination in patients without bowel obstruction might be within normal limits. Although ultrasound is readily available and thus constitutes the initial imaging modality, computed tomography (CT) of the abdomen is the imaging modality of choice [4].

Intraoperative assessment of the bowel perfusion with the need for bowel resection due to irreversible ischaemic changes is of utmost importance in the management of incarcerated hernias. The observation of bowel colour and the presence of peristalsis constitute the most common means of judging intestinal viability. These observational features however, can be very subjective and might not be readily reproducible [5]. Intraoperative fluorescence angiography with ICG provides an objective and reproducible means of assessing and documenting intestinal perfusion.

## Case presentation

**Fig. 1:**
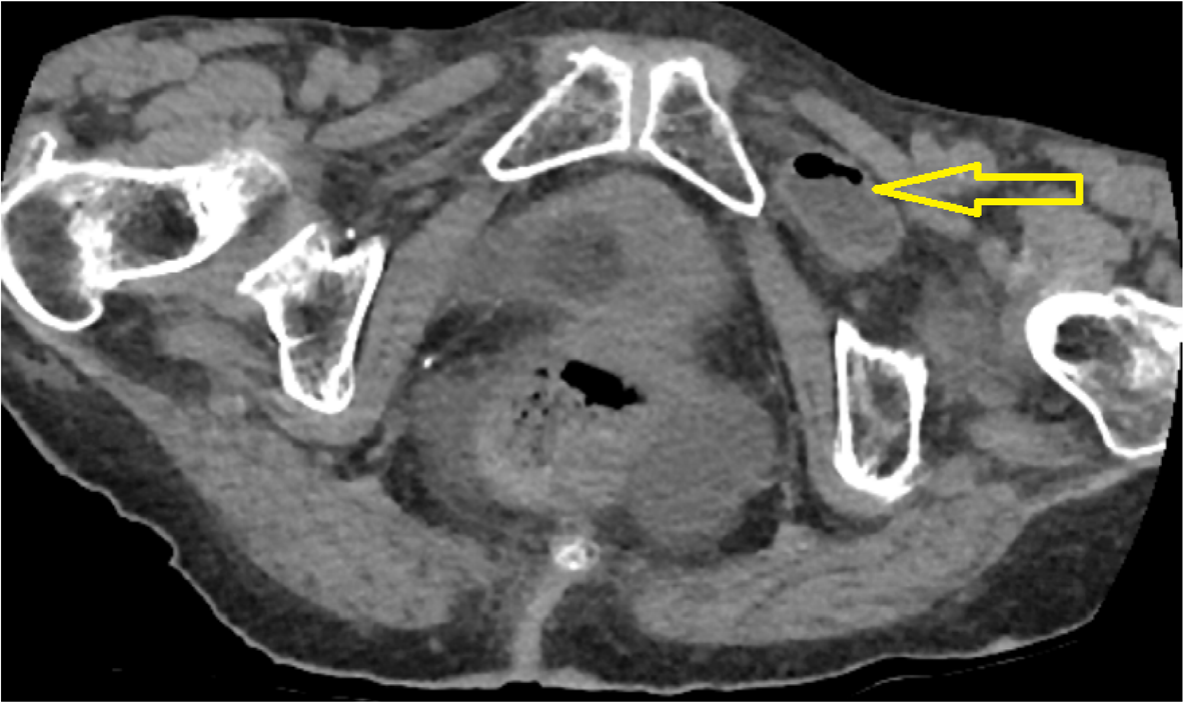
Transversal CT scan. The yellow arrow indicates bowel in the obturator foramen

**Fig. 2:**
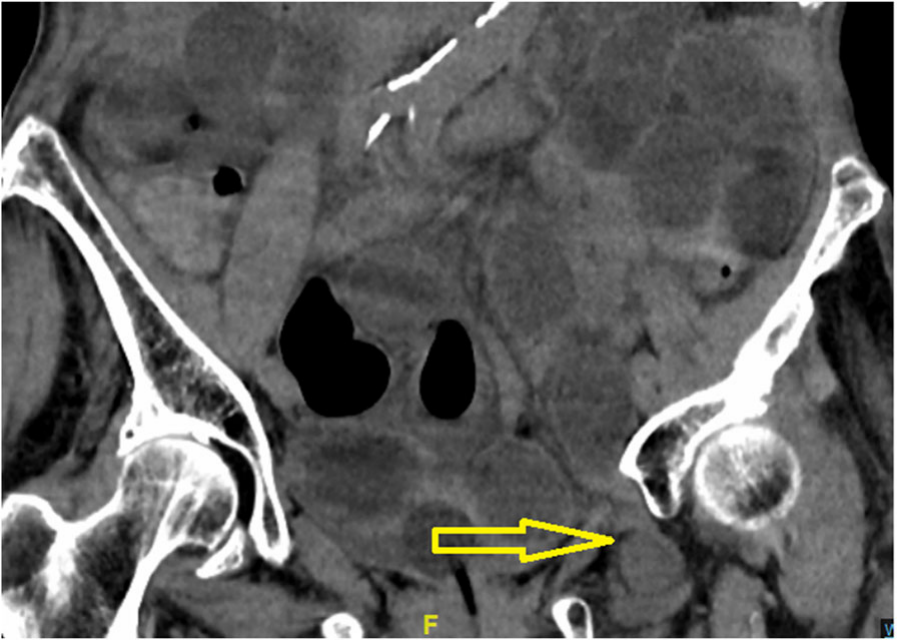
Coronary CT scan. The yellow arrow indicates bowel in the obturator foramen

**Fig. 3:**
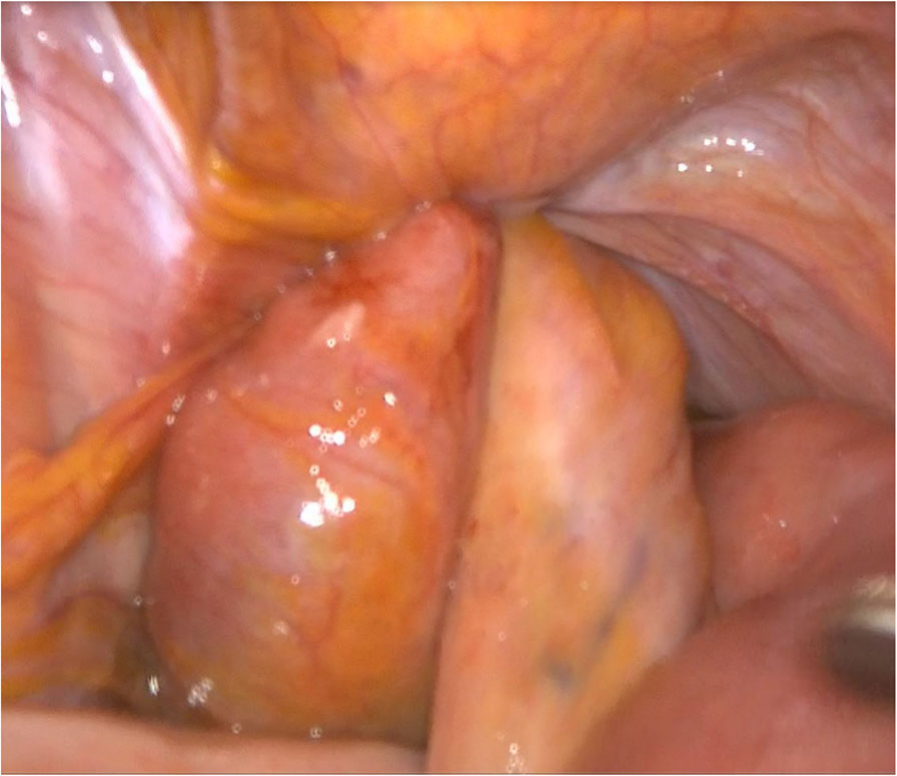
Intraoperative finding. A segment of the small bowel was trapped in the obturator canal

**Fig. 4:**
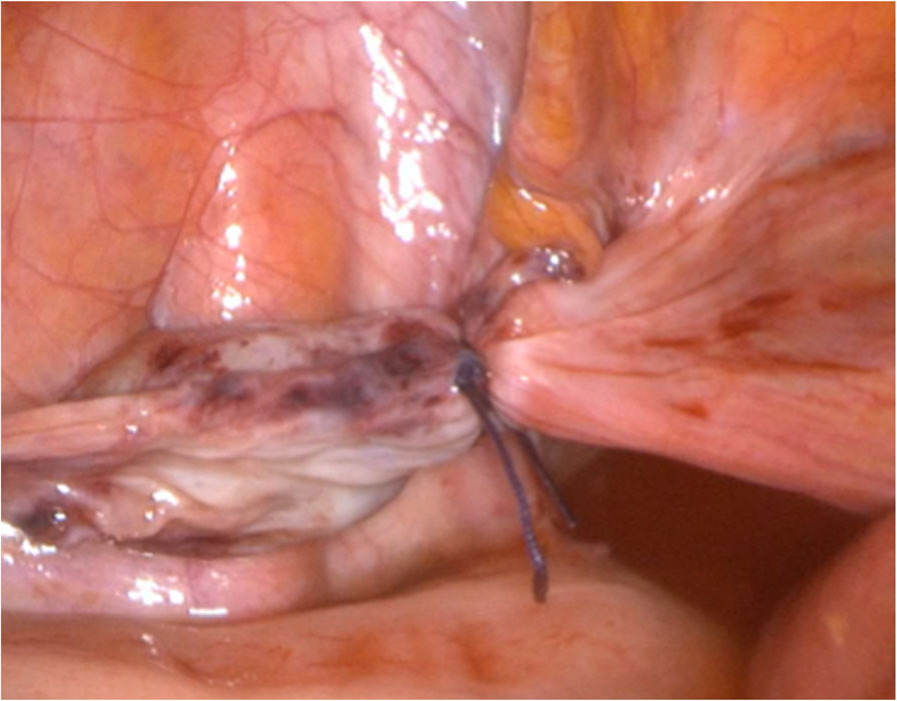
Laparoscopic management of the obturator hernia using an endoloop

**Fig. 5:**
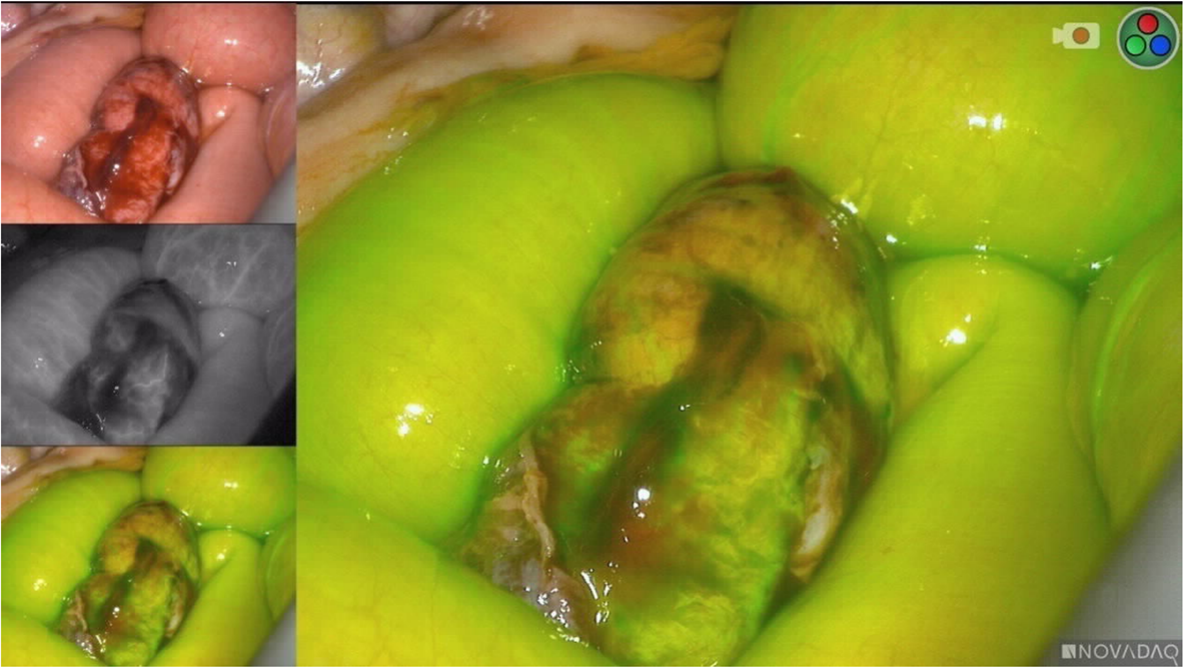
Intraoperative real-time ICG fluorescence angiography following bowel reduction. The left upper image demonstrates the laparoscopic mode; the middle image indicates the angiographic mode while the lower image indicates the fluorescence mode (corresponding to the enlarged image). Note the longitudinal discolouration in the middle of the anti-mesenteric bowel surface with lack of perfusion

**Fig. 6:**
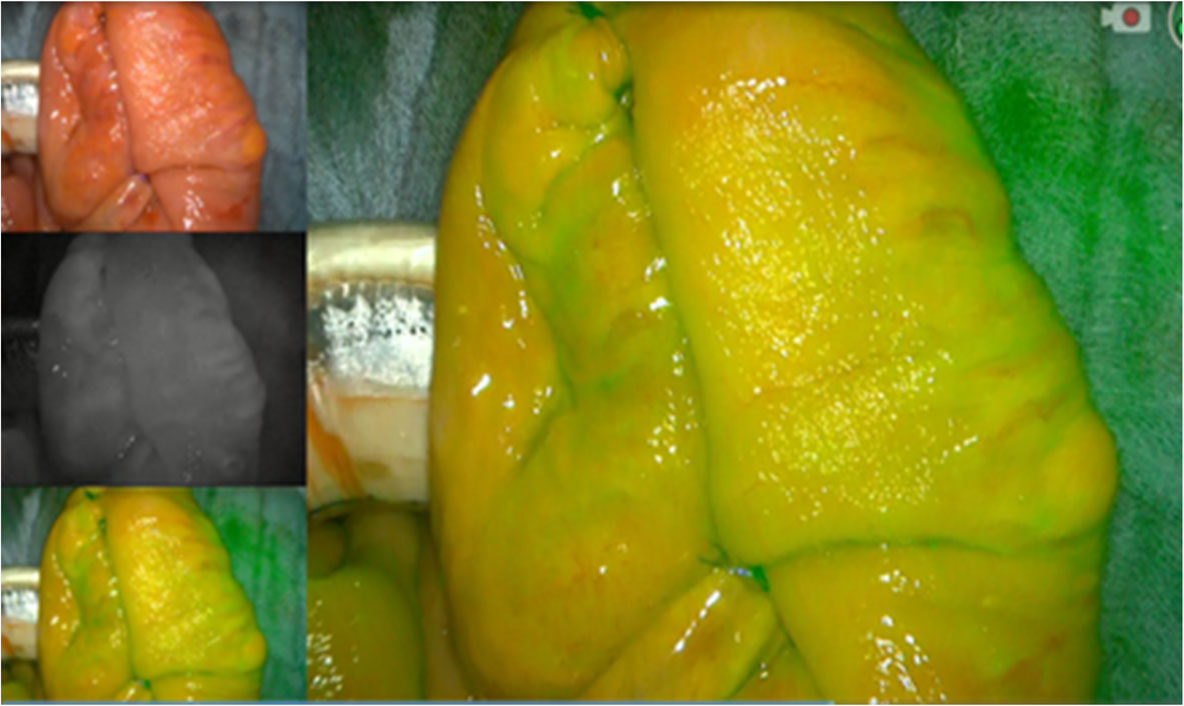
Real-Time ICG fluorescence image following anastomosis showing a well perfused anastomosis

A 92-year-old caucasian female with no previous history of abdominal surgery was admitted to our department with diffuse abdominal pain and vomiting. Physical examination revealed abdominal distension with muscle rigidity and absent peristalsis. Examination of her groins did not reveal any swelling. Bowel decompression via a nasogastric tube revealed small bowel content. A plain x-ray of the abdomen showed multiple dilated loops of small intestines. A CT of the abdomen identified the cause of the small bowel obstruction to be a herniation of the ileum between the internal and external obturator muscles (obturator foramen) with signs of incarceration, Figs. 1 and 2. An emergency diagnostic laparoscopy was performed and the trapped ileum segment was bluntly reduced, Fig. 3. The hernia was laparoscopically managed via resection and closure of the redundant peritoneum over the obturator foramen using an endoloop (Fig. 4.). The reduced bowel appeared sufficiently perfused with merely a serosal laceration. Intraoperative ICG fluorescence angiography was performed following injection of 3 ml of ICG and the bowel perfusion was observed using the PINPOINT ® system (PINPOINT; Novadaq, Canada). ICG fluorescence suggested the presence of irreversible ischaemia with the need of bowel resection, Fig. 5. The ischaemic ileum segment was resected followed by a side to side stapled anastomosis. Figure 6 demonstrates a normal bowel perfusion after ICG following anastomosis. Postoperative management included fast tract rehabilitation, physical therapy and mobilisation. The patient was discharged on postoperative day seven after an uneventful postoperative course with normal bowel movement.

## Discussion

The case of a 92 – year old female patient presenting with bowel obstruction secondary to an incarcerated obturator hernia is presented. The diagnosis was confirmed following abdominal CT. Diagnostic laparoscopy was performed and the trapped small bowel loop was reduced. Real-time ICG fluorescence angiography indicated ischaemic changes of the trapped loop warranting resection.

Traditionally, laparotomy has been considered the most common treatment option [3]. However, the laparoscopic approach has emerged as the surgical treatment of choice due to reduced hospital stay and lower incidence of postoperative complications [5–8]. Hunt et al. reported minimal morbidity and no mortality in a series of 10 patients undergoing laparoscopic surgery for incarcerated obturator hernia [6]. In a 15 - year analysis including 36 cases with obturator hernia by Ng et al. no relevant morbidity or mortality was reported in 16 patients managed with the laparoscopic approach [7]. These data confirm the safety and efficacy of the laparoscopic approach in the management of this rare hernia. Besides the well established advantages of minimal invasive surgery, the laparoscopic approach enables the detection of a simultaneous contralateral obturator hernia. This notion is in line with a recently published study by Kohga et al. [8].

Intraoperative evaluation of the intestinal perfusion with the need for bowel resection in case of irreversible ischaemic changes is of utmost importance in the management of incarcerated hernia. Judging the viability of the bowel segment in question might not always be easy. Intestinal coloration and the presence or absences of peristalsis are commonly used to judge bowel viability [9]. Both parameters however, are neither objective nor reproducible.

Real-time fluorescence angiography with ICG on the other hand is a reliable and objective means of assessing intestinal blood flow. This technique has been extensively employed in the evaluation of anastomotic perfusion in colorectal surgery [10–12]. Besides, the application of ICG fluorescence angiography has so far been employed in laparoscopic surgery, providing the surgeon with valuable information regarding perfusion and anatomy [1, 12, 13].

ICG can be injected intravenously and the presence of intestinal blood flow can be visualised using a laser beam or a near-infrared camera as it becomes fluorescent [9, 11].

## Conclusion

Obturator hernia is a very rare entity with a high risk of bowel incarceration. The laparoscopic approach should be favoured where necessary. Intraoperative ICG fluorescence angiography might be helpful in discrimination viable from ischaemic bowel thereby facilitation the decision for or against bowel resection.

## Abbreviations

CT: Computed Tomography
ICG: Indocyanine green
